# Supplementary material for: Biological volume EM with focused Ga ion beam depends on formation of radiation-resistant Ga-rich layer at block face
Source: bioRxiv. 2025 Mar 12:2024.09.16.613321. Preprint. [Version 5] doi: 10.1101/2024.09.16.613321 (PMC11722218; doi:10.1101/2024.09.16.613321)
Supplement: Supplement 1 [file media-1.pdf]

Biological volume EM with focused Ga ion beam depends on formation of radiation-resistant Ga-rich layer at block face

Zijie Yang[1], Joshua Kim[1], Cameron M. Baenen[1], Samuel J. Fulton[1], Guofeng Zhang[1], Xiaobing Chen[2], Maria A. Aronova[1], Richard D. Leapman[1*]

[*]Corresponding Author

Supplemental Information

Supplemental Note 1: Estimation of composition of surface layer from STEM high-angle annular dark-field (HAADF, elastic scattering) images of sectioned FIB-SEM Epon (epoxy resin) block.

The known composition of Epon used to prepare the samples used in this work is given in Supplemental Table 1. The cured Epon/Araldite resin has C, N, O and H atoms in the ratios $C_{42}O_{9.9}N_{0.1}H_{64}$, where the ratios represent the number of atoms per nm^3 (Supplemental Table 2). To estimate the surface layer composition, we are unable to consider H atoms because their contribution towards the elastic scattering signal is small, and most will be lost due to interactions with the Ga and/or the electron beam. We also notice that the EELS from the surface layer contains very little oxygen, which is presumably mainly lost either from interaction with the Ga^+ ion beam or the electron beam in the 1.5 keV FIB-SEM and/or in the 300 keV TEM/STEM used for the EELS analysis. We write the C atom elastic scattering cross section as $(\sigma_e)_C$ and the Ga atom elastic cross section as $(\sigma_e)_{Ga}$. We also write the number of C atoms per unit volume in the undamaged Araldite-Epon as $(n_C)_{Epon}$; the number of C atoms per unit volume remaining in the damaged Araldite-Epon surface layer as $(n_C)_{SL}$; and the number of Ga atoms per unit volume that are implanted into the surface layer as $(n_{Ga})_{SL}$. We now make use of the line profile across the block face (Fig. 4b) to determine the ratio $\mathcal{R}$ of the DF intensities at the surface layer of the block to the HAADF intensity far inside the block.

This gives:

$$\mathcal{R} = \frac{(\sigma_e)_{Ga}(n_{Ga})_{SL} + (\sigma_e)_C(n_C)_{SL}}{[(\sigma_e)_C(n_C)_{Epon} + [(\sigma_e)_O(n_O)_{Epon}]}$$

from which an expression for the surface composition can be obtained

$$\frac{(n_{Ga})_{SL}}{(n_C)_{SL}} = \frac{(\sigma_e)_C}{(\sigma_e)_{Ga}}\left\{\mathcal{R}\left(\frac{(n_C)_{Epon} + \left[\frac{(\sigma_e)_O}{(\sigma_e)_C}\right](n_O)_{Epon}}{(n_C)_{SL}}\right) - 1\right\}$$

The C and Ga elastic scattering cross sections for 300-keV electrons, which we use in our FEI Tecnai TF30 TEM/STEM can be determined from Salvat et al. (2005) (Supplemental Table 3). The surface layer composition can be determined as a function of the number of carbon atoms per unit volume remaining in the surface layer relative to the number in the undamaged Epon. From Fig. 4b the ratio $\mathcal{R}$ is approximately 3.9. This provides a relationship between the gallium to carbon-plus-oxygen composition of the damaged Ga-implanted surface layer as a function of the numbers of light atoms that are lost from the specimen.

From Supplemental Table 3, $\frac{(\sigma_e)_O}{(\sigma_e)_C} = 1.52$, from which we obtain:

$$\frac{(n_{Ga})_{SL}}{(n_C)_{SL}} = \frac{(\sigma_e)_C}{(\sigma_e)_{Ga}}\left\{\mathcal{R}\left(\frac{(n_C)_{Epon} + 1.52 \times (n_O)_{Epon}}{(n_C)_{SL}}\right) - 1\right\}$$

Based on the density of Epon/Araldite ($\rho = 1.20 \pm 0.05$ g/cm^3) and the atomic compositions of its four components (Embed-812, Araldite-502, dodecenyl succinic anhydride, and benzyl-dimethylamine), 1 nm^3 of cured resin contains 42 carbon atoms, 9.9 oxygen atoms, 0.1 nitrogen atom, and 64 hydrogen atoms (Supplemental Table 2). The STEM-HAADF images and STEM-EELS data are consistent with nearly all oxygen and hydrogen atoms being lost from the surface layer of the block by interaction with the Ga ions and/or by the electron beam.

Supplemental Table 1. Composition of Epon/Araldite embedding resin used to prepare the samples. The density of the cured resin Epon/Araldite is 1.20±0.05 g/cm^3.

Component	Mass used in mixture (g)	Formula unit	Molecular weight
EMbed 812	27.5	$C_{12}O_6H_{20}$	260
Araldite 502	17.0	$C_{18}O_2H_{19}$	267
Dodecenyl succinic anhydride (DDSA)	55.0	$C_{16}O_3H_{19}$	266
Benzyl dimethylamine (BDMA)	2.15	C_9NH_{13}	135

Supplemental Table 2. Number density of elements in Epon/Araldite embedding resin, containing four chemical components (Embed 812, Araldite 502, DDSA, and BDMA), based on a resin density of 1.20 g/cm^3.

Element	Symbol in Supplemental Note 2	Number density (atoms/nm^3)
**C**	n_{C}	**42.0**
**N**	n_{N}	**0.1**
**O**	n_{O}	**9.9**
**H**	n_{H}	**63.8**

Supplemental Table 3. Elastic scattering cross section per atom (σ_e) for E_0 = 300 keV electrons collected by HAADF detector with inner angle $\alpha_{min} = 10^{-2}$ rad, and outer angle $\alpha_{max} = 5 \times 10^{-2}$ rad. Data taken from Jablonski et al.[21]. The cross sections are given in units of the Bohr radius squared, $a_0^2 = 2.800 \times 10^{-21}$ m^2.

Element	Symbol in Supplemental Note 1	Elastic scattering cross section per atom (σ_e) (units of a_0^2)
C	$(\sigma_e)_C$	6.01 x 10^{-3}
N	$(\sigma_e)_N$	7.70 x 10^{-3}
O	$(\sigma_e)_O$	9.13 x 10^{-3}
Ga	$(\sigma_e)_{Ga}$	67.2 x 10^{-3}

Supplemental Figure 1

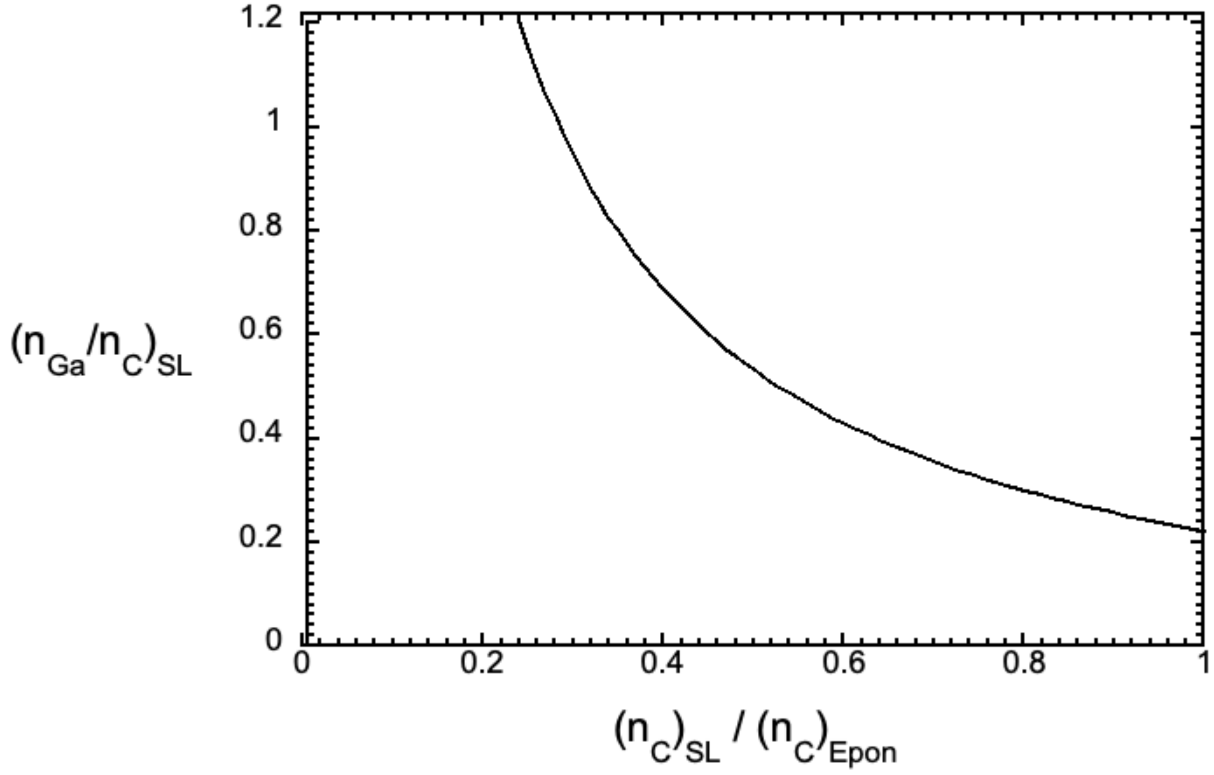

Supplemental Figure 1 | Composition expressed as gallium to carbon atomic ratio for block surface layer (SL) , as a function of the ratio of light atoms remaining in the surface layer relative to those in the undamaged Epon/Araldite resin, based on HAADF images and elastic scattering cross sections for C, O, and Ga. The almost complete absence of oxygen in the EELS spectra from the surface layer indicates that most of the oxygen is lost from the Epon after exposure to the Ga beam and/or the electron beam. It is also likely that some of the carbon atoms are lost from the surface layer, as well as most of the hydrogen atoms which are known to be removed at a low electron fluence. If none of the C atoms are lost from the Ga-implanted layer, the gallium to carbon ratio in the surface layer is estimated to be 0.22:1. However, if only 50% of the C atoms remain in the surface layer, the Ga to C atomic ratio is 0.53 to 1, or 35 atomic % of gallium.

Supplemental Note 2:

The atomic ratio of Ga:C in the surface layer (*SL*) of the block $\left(\frac{N_{Ga}}{N_C}\right)_{SL}$ is proportional to the ratio of integrated core-edge signals in energy loss window Δ = 100 eV, above the respective core edges, $\left(\frac{I_{Ga}(\Delta,\alpha)}{I_C(\Delta,\alpha)}\right)_{SL}$ after inverse power law background subtraction, and α = 10 mrad is the EELS collection semi-angle. The ratio of core signals is then multiplied by the inverse ratio of the core-edge cross sections for the same energy range Δ and collection semi-angle α, $\left(\frac{\sigma_C(\Delta,\alpha)}{\sigma_{Ga}(\Delta,\alpha)}\right)$ to obtain the atomic ratios, in the surface layer:

$$\left(\frac{N_{Ga}}{N_C}\right)_{SL} = \left(\frac{I_{Ga}(\Delta,\alpha)}{I_C(\Delta,\alpha)}\right)_{SL} \cdot \left(\frac{\sigma_C(\Delta,\alpha)}{\sigma_{Ga}(\Delta,\alpha)}\right)$$

Due to the large energy loss difference between the C K edge (285 eV) and the Ga $L_{2,3}$ edge (1,140 eV), and uncertainly in the Ga cross section near the threshold energy, as well as some uncertainty in the EELS collection semi-angle α, we make use of a standard reference spectrum from gallium oxide (Ga_2O_3) that we have acquired. We can then write:

$$\left(\frac{N_{Ga}}{N_O}\right)_{Ga_2O_3} = \left(\frac{I_{Ga}(\Delta,\alpha)}{I_O(\Delta,\alpha)}\right)_{Ga_2O_3} \cdot \left(\frac{\sigma_O(\Delta,\alpha)}{\sigma_{Ga}(\Delta,\alpha)}\right)$$

Then substituting the identity,

$$\left(\frac{\sigma_C(\Delta,\alpha)}{\sigma_{Ga}(\Delta,\alpha)}\right) = \left(\frac{\sigma_C(\Delta,\alpha)}{\sigma_O(\Delta,\alpha)}\right) \cdot \left(\frac{\sigma_O(\Delta,\alpha)}{\sigma_{Ga}(\Delta,\alpha)}\right)$$

We find that

$$\left(\frac{N_{Ga}}{N_C}\right)_{SL} = \left(\frac{I_{Ga}(\Delta,\alpha)}{I_C(\Delta,\alpha)}\right)_{SL} \cdot \left(\frac{\sigma_C(\Delta,\alpha)}{\sigma_O(\Delta,\alpha)}\right) \cdot \left(\frac{N_{Ga}}{N_O}\right)_{Ga_2O_3} \cdot \left(\frac{I_O(\Delta,\alpha)}{I_{Ga}(\Delta,\alpha)}\right)_{Ga_2O_3}$$

The ratio of $\sigma_C(\Delta)$: $\sigma_O(\Delta)$ for a primary beam energy of 300 keV and a window of Δ = 100 eV above the C and O K-edges is 3.56:1 from the Gatan DM/EELS software (Supplemental Figure 2). The ratio of Ga:O in gallium oxide is 2:3, so that $\left(\frac{\sigma_C(\Delta,\alpha)}{\sigma_O(\Delta,\alpha)}\right) \cdot \left(\frac{N_{Ga}}{N_O}\right)_{Ga_2O_3}$ = 2.39.

Therefore:

$$\left(\frac{N_{Ga}}{N_C}\right)_{SL} = \left(\frac{I_{Ga}(\Delta,\alpha)}{I_C(\Delta,\alpha)}\right)_{SL} \cdot \left(\frac{I_O(\Delta,\alpha)}{I_{Ga}(\Delta,\alpha)}\right)_{Ga_2O_3} \times 2.39$$

Supplemental Figure 2

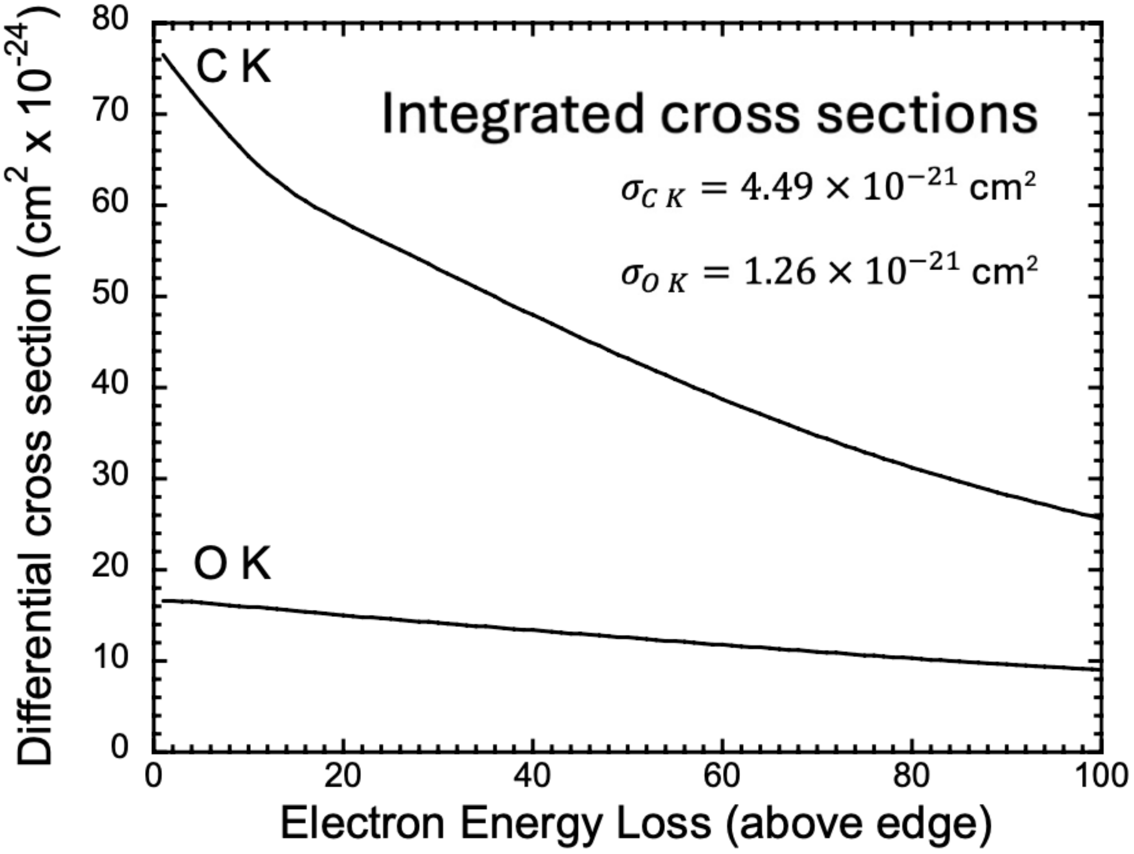

Supplemental Figure 2 | Integrated EELS C K and O K edge cross sections per atom from the core edge threshold energies of 285 eV and 532 eV, respectively, up to Δ = 100 eV above the edges, for a collection semi-angle of α = 10 mrad. Since the two edges are relatively close in energy, the ratio of cross sections for carbon to oxygen $(\sigma_C\,(\Delta, \alpha))/(\sigma_O\,(\Delta, \alpha))$ computed from the Digital Micrograph EELS analysis module will be less sensitive to uncertainties in the collection semi-angle α.

Supplemental Figure 3

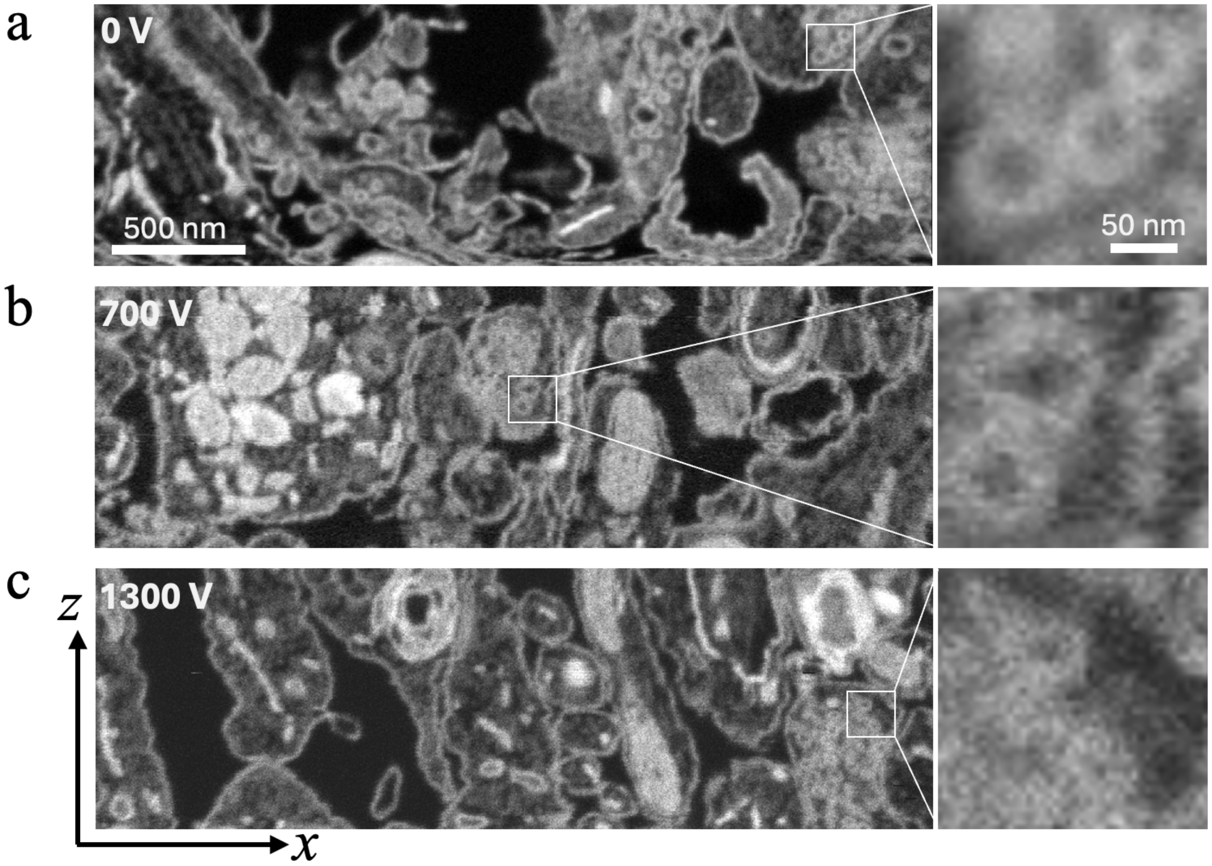

Supplemental Figure 3 | Effect of energy selected backscattered grid voltage on z-resolution in FIB-SEM images. Orthoslices in x-z plane for ESB detector grid voltage of 0 V (**a**); grid voltage of 700 V (**b**); and grid voltage of 1300 V (**c**). Clusters of synaptic vesicles indicated by boxes are visible regardless of grid voltage, with each vesicle containing a well-defined lumen surrounded by a membrane of width ~10 nm (expanded views of boxed regions). These images show that applying a grid voltage in front of the ESB detector to reject backscattered electrons, which have suffered inelastic scattering events deeper in the specimen block, does not account for high z-resolution in planes perpendicular to the block-face.

Supplemental Movie 1 | 3D image of presynaptic terminal obtained from a sample of cultured neurons in the FIB-SEM. 3D data set was acquired in a Zeiss Crossbeam 550 FIB-SEM operating at a primary electron beam energy of 1.5 keV and probe current of 2 nA. The sample was prepared following the UC San Diego NCMIR protocol, as described by Deerinck et al.[26]. The sample was milled with a 30 keV Ga^+ beam, current of 700 pA, pixel dwell time of 16 μs and a pixel size of 3 nm in x, y and z. A cluster of synaptic vesicles in evident in the animation, each with a hollow lumen and outer diameter of ~40 nm. The data illustrate that features are discerned on a scale of 5-10 nm, which would not be distinguishable without the high surface layer concentration of implanted Ga atoms, and the ability to acquire electron images at an electron fluence of $\sim 10^4$ electrons/nm^2.
